# Anonymization, accountability, and access: legal dimensions of health data sharing in federated networks. Perspectives from empirical study

**DOI:** 10.3389/fdgth.2026.1719728

**Published:** 2026-03-19

**Authors:** Magdalena Kogut-Czarkowska, Mahsa Shabani

**Affiliations:** 1 Faculty of Law and Criminology, Ghent University, Ghent, Belgium; 2 Timelex BV, Brussels, Belgium; 3 Faculty of Law, Law Center for Health and Life, University of Amsterdam, Amsterdam, Netherlands

**Keywords:** data protection, EHDS, federated networks, GDPR, health data, secondary use

## Abstract

This paper explores the perspectives of stakeholders involved in federated networks for health data sharing, focusing on the legal and practical dimensions of data protection and governance under GDPR and EHDS in the development of such infrastructures. Using a qualitative approach centered on perspectives of 19 experts with experience in projects building federated networks, it investigates the perceived challenges in fulfilling specific obligations under the GDPR, as well as in establishing the contractual framework of a federated network, including the arrangements and mechanisms required to control data access and to define the conditions for lawful and effective data sharing and reuse. The study critically assesses the commonly cited assurance that “data never leaves the node” and evaluates the compatibility of federated approaches with data protection requirements. It highlights key tensions between legal theory and practical implementation, offering insights relevant to the design and governance of other federated architectures and emerging European data spaces. Study results indicate that while the advantages of a federated approach, such as data minimization, should not be discarded, there are also significant challenges of aligning federated networks architectures with data protection requirements in particular. Federated networks help initiate discussions about data sharing with new data holders, but they do not offer a straightforward solution to legal and technical challenges of data sharing.

## 1 Introduction

Sharing health data through federated networks has been promoted as a more secure and legally compliant approach to enabling biomedical research (1, 2). In academic writing, “federated network” is not used consistently and often overlaps with terms like “federated repository”, “federated data sharing”, “federated research network”, and “federated infrastructure” (3). This paper adopts the same interchangeable use of terminology. In this model, health data remains within the infrastructure (node) of the data holder (also referred to as a custodian) while the federated infrastructure allows data users to query the data (via federated analytics) or use it for training their artificial intelligence (AI) models (via federated learning) (4). A federated network of nodes is typically supported by a central infrastructure and may offer a unified interface that allows users to search and access data across nodes as if it were a single database.

Federated networks have been seen as enablers of collaborative research across distributed datasets without requiring the data to leave its source (5–7). At the same time, by design, they involve multiple stakeholders collaborating across institutional and sometimes national boundaries without centralizing data (3). Consequently, this decentralization leads to unique legal and organizational questions (8) under the General Data Protection Regulation (GDPR) (9) and other applicable regulations.

The GDPR constitutes the horizontal EU legal framework for the protection and processing of personal data. Article 89 (1) GDPR requires that when using personal data for scientific research, appropriate safeguards, such as anonymization or pseudonymization, must be put in place to ensure data minimization. The choice of minimization measure has significant implications: when data is pseudonymous, the GDPR requirements still apply (10, 11), whereas non-personal (anonymous) data is not regulated by the GDPR. Moreover, if the data processed within the federated network is pseudonymized, network participants must assess whether they act as controllers or processors for each processing operation. This assessment can trigger the need for a contractual arrangement to clearly define their respective roles and responsibilities (8). Furthermore, to achieve its aim of enabling privacy-compliant data reuse, the federated network must adopt clear rules and mechanisms governing access to data made available in the nodes. Consequently, the key considerations for establishing a GDPR compliant federated network, include, but are not limited to, ensuring proper minimization of the data in the nodes, clearly defining roles and responsibilities of the stakeholders (12, 13) and establishing robust data governance frameworks enabling data re-use (14, 15).

Previous studies such as a report published in 2017 by Knowledge Exchange (16) on the evolving landscape of federated research data infrastructures, reported experts views from various organizations operating such infrastructures in their respective countries. That report was not limited to a specific research domain and addressed a broad spectrum of topics, including definitions of federated infrastructures, their operational models, as well as the associated challenges and drivers. Since then, a number of guidelines (17) and resources (18, 19)^,^ have been published on the development of “data spaces,” including those incorporating data federation. However, these documents tend to offer high-level guidance and often lack detailed, practical insights. In parallel, case study articles have examined specific projects involving the establishment of federated data-sharing frameworks, typically within a single initiative or domain (20–22). To our knowledge, our study is the first to empirically examine the practical challenges of sharing health data through a federated network, with particular attention to the legal constraints and requirements under the GDPR. It aims to address this gap by offering insights into the perspectives of both users and developers of federated repositories designed for health data sharing in a research context.

## 2 Materials and methods

### 2.1 Study design

We used a qualitative research design consisting of conducting individual semi-structured interviews.

### 2.2 Data collection

The study team developed a semi-structured interview guide. The interview guide was pilot-tested for content and understandability and adapted to improve flow. The interviews took 45–90 min and were conducted in English online and recorded via Teams. Subsequently they were transcribed ad verbatim, pseudonymized and coded. New interviews were conducted until data saturation was reached. All interviews were conducted between March 2024 and April 2025. The study was approved by the Ethical Committee at the Faculty of Law and Criminology at Ghent University.

### 2.3 Research team

The research team consisted of MKC and MS. The researchers designed the interview guide (submitted as supplemental material). The interviews were all conducted by MKC, a female PhD candidate, with the support of MS for some interviews. Transcripts were coded by both members of the research team.

### 2.4 Participants and recruitment

We conducted interviews with 19 experts who had experience in participating in or developing such federated repositories The participants represented diverse fields, including law, ethics, project management, data science, infrastructure development, AI, and medicine. Overview of the experts backgrounds is provided in supplemental material. Most of the interviews were one-to-one. For one interview, two participants from the same institution requested to be interviewed together, which was accommodated. To qualify for participation in this study, individuals needed to meet the criterium of designing or implementing data sharing solutions based on federated approach, demonstrated through practical experience. They also needed to demonstrate working knowledge of English. Given the specificity and limited accessibility of the target population, purposive and snowball sampling techniques were employed to identify suitable participants, in particular targeting experts who had participated in EU funded projects, such as Horizon Europe or Horizon 2020.

Contact information was retrieved from an Internet search or obtained via the researcher's network. After inventorying potential participants, they were approached through email and/or LinkedIn. Before the interview started, the participants received an email containing an information letter. After agreeing to participate, they received another email containing additional information and consent form.

### 2.5 Analysis

To analyze the data, we relied primarily on thematic analysis. We performed an inductive content analysis, where the content categories are derived from the data, rather than pre-determined. Transcripts were coded into narrow content categories. The themes identified in the study and discussed in this paper are provided as supplementary Material.

### 2.6 Use of AI

During the manuscript preparation process, the authors used OpenAI's ChatGPT, version GPT-4o, August 2025 release (accessible via https://chat.openai.com), to assist with language editing and refinement. The tool was employed to improve sentence clarity, restructure complex formulations, and ensure consistent academic tone. All outputs generated by the AI were critically reviewed and edited by the authors to maintain accuracy and integrity of the content.

## 3 Results

Our findings depict perceived challenges in meeting specific GDPR obligations and in establishing contractual framework of a federated network, arrangements and mechanisms for controlling data access and conditions necessary for lawful and effective data sharing and reuse.

### 3.1 Federated networks under the lens of the GDPR

In the interviews, experts shared their experiences on the applicability of GDPR and national laws to data processing in federated networks, compliance with ethical requirements, and the assessment of GDPR roles of the participants in the data processing.

#### 3.1.1 Applicability of GDPR rules and other national laws

Discussions about implementing a federated approach often arise in response to concerns about GDPR compliance in sharing sensitive health data, as noted by one of the participants. They described it as “*a conversation about comfort” (P016)*, emphasizing the perceived reassurance that comes from data not leaving the local infrastructure. However, they warned that federated processing does not resolve all legal challenges. Experts agreed that the fact that the data does not move does not mean that data processing falls outside the scope of the GDPR:

P008: “So data doesn't move, so there's no legal issues”. And especially in the GDPR context, we know that that's a gross simplification. So I was very skeptical of this idea.

Participants also stressed that the fact that data remains in the nodes does not mean that it is not being processed. They pointed out that if data is used for research, even if researchers are not accessing the data and querying it remotely, this data is still processed at its location (*P014:* “*there is some processing of personal data going on somewhere”*). Hence, they reflected that even if there is no data transfer, when personal data is being processed, the requirements stemming from GDPR apply, such as requirement to demonstrate a legal basis.

Moreover, participants observed that when the data federation connects nodes established in various countries, then additionally the laws of those countries have to be considered.

P003: So we can imagine a project where we will have like I don't know, let's say 5 different institutions from different countries in Europe. So that means that we will have in the end five different legal frameworks that we will have to take into account, right?

In particular, they stressed that those national laws are relevant for determining the conditions of the processing of the data stored in each node and that they apply despite GDPR harmonizing data processing rules in the EU.

#### 3.1.2 Diverging rules on obtaining ethics approvals

Many participants highlighted that national laws have diverging rules on the legal requirements and ethical review processes for the use of patient's health data for research purposes. Consequently, individual data nodes need to obtain assistance from their national legal experts. Several interviewees reported that administrative and legal bottlenecks hindered the nodes from joining the federation.

Participants reported difficulties in obtaining ethical approvals, attributing these challenges to ethics committees' limited understanding of federated data sharing. Another issue concerned the requirement to specify the nature of data processing in the ethics application, which is typically submitted at an early stage of the project, when technical details are still evolving. One participant noted that the approval stated AI developers would not access the data and this assumption later proved problematic during the implementation of the federated network.

P008: But as soon as you say “data never leaves” then you start being all of these. .. you start coming back and be like “well you need to move some data here temporarily” and then you start saying “well we need to be able to see, it'll be great if I could just see some data of your data to know what you have” or “to be able to see if it's worth me applying for your data” and all of these things. Like if you start with this guarantee of data never moves, you'd start to get into trouble.

#### 3.1.3 Establishing GDPR roles of the federated networks participants

There were conflicting opinions on which organization is the controller of the data processed in the federated network during the research activities. While data holders were generally viewed as original controllers of the data that they make available through the network, there were questions around the position of the operator of the central infrastructure of the federated network and the other data users (researchers). One expert viewed the operator as the controller, while others took the position that the operator plays a processor role. The participants also struggled with qualification of the role of the users of the data because when the data is made available to them in a secure environment, with technical and organizational measures applied, they considered this data to be anonymous for the user. Moreover, one of the participants warned of challenges in taking the approach that the user is a controller and thus needs to demonstrate their legal basis for processing. They questioned whether assigning a controller role to the user improves the level of data protection:

P008: (…) it doesn't help much if the user has to come with their own legal basis, from their own context to analyze data that they can't see or have no big influence [on] the security of the data.

#### 3.1.4 Contractual framework for regulating use of data in federated networks

Participants reported mixed experiences with concluding agreements on the provision and use of health data within the federated network. Experts described a range of contractual models that had been implemented across projects. Some advocated for the use of joint controller arrangements between data-contributing partners (holders) and technical partners (users). Others noted that certain projects had opted for data processing agreements between participants, though they did not necessarily endorse this approach. Another expert emphasized that, in some cases, data sharing within a consortium can be governed solely by the consortium agreement and an agreed study protocol.

Many of the interviewees reflected on challenges in creating a contractual framework between the participants of the network and the lengthy discussions between the parties. One interviewee recalled that even in a closed consortium group, negotiation of a data sharing agreement took a long time:

P017 It had a rough start getting that actual agreement approved by the different lawyers in the different countries. (...) The problem was not with the different node, and approving them, but getting a legal document to say what, how user should be able to access that central node and the responsibility of those hosting the central node and so on. So that was an eye opener for me. I've been mostly focusing on the different repositories or nodes, not so much on the central part.

Some attributed these challenges to communication gaps between legal and technical teams, particularly when it comes to translating legal requirements into technical specifications:

P003: I'm gonna say that sometimes are too abstract, and when they are concrete, they are concrete in the legal way. That at some point there is some sort of missing piece, so it can be properly translated to technicalities.

Despite these challenges, the need for putting in an effort for developing a robust contractual framework was emphasized especially by participants working on open federated repositories, i.e., those which accept new members, beyond the original consortium of collaborators. The participants advocated for clear agreements regulating access to data and outlining what the users can and cannot do with the data. In turn, agreements with the data holders should outline the “*limitations, the restrictions for the use of data” (P006).*

### 3.2 Data anonymization in federated networks: between privacy and utility

The anonymization of health data in federated networks was a central topic discussed by nearly all interviewed experts. They highlighted challenges in determining whether data shared within the network qualifies as anonymous or still constitutes personal data. The experts also examined the trade-offs associated with overly strict anonymization practices and critically questioned the adequacy of the current regulatory framework for defining personal data.

#### 3.2.1 Anonymization in federated networks: definition and implementation

Many experts began by pointing out that the difficulty in interpreting the GDPR concepts of anonymous and pseudonymous data creates significant challenges in establishing a common understanding among network participants about when data can be considered truly anonymous.

P011: In terms of data protection issues, I mean, it's always a thing of things like, you know, what's personal data, what's pseudonymization, how that's interpreted, how that's interpreted even among countries is very different depending on your... because it's all comes down to like state-of-the-art, and that could be different, wildly different in one country to another, depending on the technological capabilities.

One participant expressed a view that while centralized repositories face similar challenges, they are amplified in federated networks. The involvement of multiple participants in the network contributes to uncertainty about which operations involve the processing of personal data, particularly when each participant has a different perception of the status of the data based on their ability to re-identify it. On the other side, federated approaches benefit from pooling together diverse experts on data anonymization, which may not be available to individual registries. Participants also pointed to initiatives aimed as development of anonymization tools. In particular, participants believed that those tools may be helpful for data holders which do not have the needed expertise inhouse:

P013 The good thing with federated infrastructure is usually they try to make also tool for harmonizing the pseudonymize and anonymization on these nodes. So for example, in many projects, like in my project they are making anonymization tools and they are providing apps for the local level for anonymizing medical images and data, and it can help a lot, because many times on a local level, many times they are lacking the knowledge and proper tools for anonymizing and pseudonymizing the data well.

#### 3.2.2 Compromising on data utility? trade-offs of data anonymization

Participants noted that conducting analytics within federated networks requires individual-level data at each node, which can seldom be fully anonymized. Many participants warned that overzealous anonymization deprives data of useful information or may even destroy the value of data. To address this, they emphasized the need to find the right balance between protecting data privacy and ensuring its usability for research. As one of the participants put it, striking the balance requires considering the specific context,:

P008: None of those things are ever determined at the output because they all come down to that kind of we've always balanced data utility and data privacy, but you can only do that very in a very contextual way, like for this study or this field, what's the right balance?

Another participant raised a broader question concerning the costs and trade-offs involved in protecting privacy through anonymization:

P001: But well, I've been told that it's usually a lot more expensive, so it's this trade off, that you can protect privacy, but it's it just comes at a certain cost and that's also a policy question. It's not just a technical question. Do we want this? How much efforts do we want to put in to have both accuracy and privacy in this model?

#### 3.2.3 Adapting regulatory guidelines on data anonymization

Several interviewees found it difficult to interpret the GDPR concepts of personal data and anonymous information in the context of health data, and questioned whether existing guidelines and case law provide enough support for applying these concepts in research via federated networks. Some advocated for a new, detailed regulatory guidance regarding anonymization. They pointed out that supervisory authorities' guidelines should engage with the realities and complexity of a federated model. To more accurately reflect reality of using personal data in medical research, some participants championed for recognition of an intermediate data category, one between anonymized and pseudonymized data. One participant referred to it as “well anonymized data”, under which it is “*P013 very hard or almost impossible to single out individuals”*. Another participant noted a concept of “functionally anonymous” data, recognized by the research community in the UK. This model takes into account not only the measures used to de-identify the personal data but also the context in which it made available and controls put in place to minimize the risk of re-identification by the researchers:

P014 And so that's kind, of it's not in the legislation anywhere, but it's in the psyche of the data research profession in the UK. So there's this concept of anonymous and suitable functional anonymity, which is a combination of pseudonymization, plus, you know, access controls, plus researcher training, plus all the other things that you have in place. [This] equals, you know, functionally anonymous data, even if the data, if it was outside of that environment would be personal data or identifiable data.

### 3.3 Establishing data governance framework in federated networks

Participants discussed their experiences with data governance in federated networks. For some, this was mainly a matter of organizational arrangements, such as defining data access models and identifying who should make decisions about access. For others, data governance was understood in more technical terms, relating to the functionalities of federated networks provided to users. This included determining whether users could directly access and view data, as well as the technical mechanisms supporting access requests and decision-making. Participants highlighted the complexity involved in establishing governance frameworks and pointed to limited availability of detailed information on existing governance models that could serve as references for new projects. Many recognized that discussions around data governance were hard to resolve and took a long time, biting well into the project timeline. As one of the participants pointed out, these decisions are “*pushed endlessly down the road (P008)*”. In a similar vein, some highlighted that technical complexities of setting up federated networks data governance are less than human complexities in reaching an agreement.

#### 3.3.1 Managing data access: local or centralized?

Many participants reflected on the tension between maintaining control by data holders (nodes) over the use of “their” data and the need to streamline access for data users. One participant noted a fundamental contradiction in federated approaches: while they aim to preserve local control over data access, their purpose is also to streamline data sharing, foster new forms of collaboration, and reduce privacy and security concerns associated with data access.

The question which stirred different opinions was on the party or parties that should be responsible for making data access decisions in a federated networks. Some participants indicated that data access decisions are always the responsibility of the data holder and pointed that this stems from expectations regarding data access through a federated networks.

P008 You can't be telling people they control their data and then tell them to delegate to Central Access Committee. The model in which data nodes are in control is also associated by participants with varying conditions between the nodes under which the data can be shared.

Other participants strongly opposed the decentralized governance model, and argued that a model relying solely on individual permissions quickly becomes very burdensome (*P013 „administrative nightmare”*) and is unsustainable. They favored an approach in which the nodes delegate though a data contribution agreement, the right to grant access permissions to a centralized governance body. Overall, their view was that there should be a designated data holder (lead controller) or a body representing the data holders which is responsible for granting access.

#### 3.3.2 Importance of harmonizing data access process

As many pointed out, a governance model based on individual permissions issued by the nodes introduces complications for data users and slows down access procedures. To address these challenges in repositories which de-centralize data governance, some proposed to standardize the decision process by developing a service to coordinate obtaining data access permissions in case of a request for data from multiple holders. Others favored alternative approaches, such as the creation of a centralized data access request protocol or the introduction of a veto model, in which nodes can refuse to execute a query rather than having to actively agree to it. Another proposed solution was a contractual arrangement under which nodes agree to follow the decision made by one of the participating nodes.

Among other mentioned elements of a well-oiled data governance framework were standardization of ethics approval process, transparency of the approval conditions and implementation of technical solutions to harmonize and streamline the process. In terms of transparency, participants emphasized the importance of providing detailed descriptions of the datasets and clearly communicating the conditions of their use to potential users in advance. Some of the suggested technical solutions included creating a shared platform where all node Data Access Committees (DACs) can access incoming requests. Organizational measures included using predefined terms outlining permitted data uses, supported by several preset levels of granularity based on privacy risk. Participants mentioned also attempts to set up tiered tracks for data access requests (fast, medium and slow), depending on their complexity. As one participant explained:

P014: So that things that are considered to be more routine, or have already in the scope of our previous decision by the Data Access Committee, and that the lead controller or whoever's responsible for the process would not then need to go to any process, whether kind of with each controller or whether with the lead organization, because they're all they're kind of within a pre-approved as a fast category. More novel things involved or things involving deceased individuals or children or, you know, rare conditions or something would be in a slower track where they go for individual scrutiny.

#### 3.3.3 Role of the data access committee (DAC)

Many participants highlighted the role of a DAC, exploring ways in which it could be set up within a federated repository, the functions it might perform, and its potential to streamline timely responses to data access requests. As one of the participants shared:

P013 But I would say in efficient good project there should be a data access committee on a federated level, which is a person or a committee which can which can quickly react to the to the applicants, to share the data.

The DAC, as noted by some participants, can be set up individually by each node, or on a central level by the lead controller or by the repository operator, together with representatives from the nodes, the general public and patients. One of the participants pointed to weighing whether the data holders with larger datasets should have more say in the decisions on data access set up. Participants underlined that the role of the DAC is to verify the data request against legal and ethical conditions for access. Some of the participants argued that the optimal model for a speedy data governance is when the decisions on access are fully delegated to a central DAC. One participant explained that delegation means that the nodes “*P014 effectively give permission within certain guardrails for data access to be operated without recourse back to the contributing party each time.*” While most of the participants agreed that delegating decisions to DAC has benefits, some point out that in practice it may not be acceptable to the data holders because of their internal policies. One participant reflected:

P016 Yes. Yeah. I mean we've tried to put policies in place where there is a controller agreed for a particular access...for a particular processing action, and that we've got some agreement that, you know, that one controller can represent others, but it still falls down when there is an institutional requirement to review, the need to review any request.

Some argued that the DAC should serve only as an advisor, with final decisions on data access or algorithm approval resting with the nodes or a federated networks steering committee. As one participant underlined that while an advisory model may appear less streamlined, DAC recommendations are typically followed in practice. Others highlighted the evolving role of DACs in contemporary research, stressing the need to assess algorithms which are to be trained on the data with the same level of scrutiny as data access conditions.

#### 3.3.4 Data holders, central aggregator and data users: defining the roles and tasks in federated networks

Experts also discussed their understanding of the tasks of the parties involved in the federated repository. They pointed to changing role of the data holders, which not only host the data and decide on making their data available for research, but effectively become compute service providers for the researchers (data users). Nodes may also need to make decisions on which results of the queries they release to the researchers. According to interviewees, the repository operator, sometimes called the central aggregator, was tasked with various responsibilities as it served as an intermediary between data holders and data users. Tasks mentioned for the operator in its role as an intermediary included: holding a centralized data catalogue, facilitating the contact between the potential users and data holders, signing contracts with new data holders and onboarding them, supporting the node custodians, advising them on the set up of their node, setting up the conditions to become a node and verifying them. On the latter point, a few participants mentioned that the operator should impose security requirements for the data nodes. Other listed tasks of the operator included: harmonizing the data access processes, optimizing the data in the repository and bringing visibility to the positive results of the federated research. Some stressed that types of responsibilities assigned to the central operator impact the qualification of its role (if any) with respect to shared data, as defined by the GDPR. The role of the data users in the federated network was rarely discussed in detail, however one participant pointed that, when publishing results of the research, the users should be obligated to acknowledge the network through which the access to data was obtained.

Overall, the participants acknowledged that mixed models of data governance can be implemented, recognizing different levels of data federation. They stressed that the governance model can also evolve from a very simple one in a federation between members of a project consortium, to a more complex model when the federated repository can be joined by new, external members. According to one participant, the consortium developing a federated network initially decided not to create a data governance model, since the approved research protocol already specified how the data could be used and by which partners. This is likely to change, however, when the network expands to include new members and research projects.

## 4 Discussion

### 4.1 Are federated networks opening the door to (even more) GDPR compliance discussions?

Promoting the use of secure, federated data infrastructures for cross-border health data exchange was a key objective integrated into the 2020 European strategy for data (23), paving the way for the European Health Data Space (EHDS) (24). In parallel, projects and initiatives focused on building federated repositories have gained significant momentum. This trend has been driven by advances in privacy-enhancing technologies (PETs), such as federated learning (25), which aim to address privacy risks through technical solutions and support compliance with the GDPR. Against this backdrop, federated approaches have been perceived to be aligned with principles of data minimization, accountability and purpose limitation, as the data remains within the data holder's infrastructure and is not transferred to external parties (26). However, our study corroborates earlier observations (15) that federated networks cannot be perceived as a silver bullet for avoiding legal challenges. The findings indicate that the possibility of sharing data in a federated manner, rather than through centralized data pooling, can serve as a valuable entry point for initiating dialogue with potential data providers seeking alternative, GDPR-compliant approaches to data sharing. However, opening this conversation can be a double-edged sword. While a federated approach may enable the participation of new data holders in scientific projects, participants shared that establishing the legal framework governing such repositories presents significant challenges. These stem from the inherent complexity of the technical architecture and the involvement of data holders from multiple jurisdictions, which are subject to different national rules. This often leads to prolonged discussions between legal and technical teams.

Our study supports previous findings that, during the implementation phase, federated networks encounter a wide range of data use conditions and restrictions imposed by individual data holders (27). Additionally, many data providers encounter difficulties in obtaining ethics approvals and compiling the required documentation. The study results suggests that these issues can arise due to a lack of understanding of the federated model by ethics committees, the absence of a clearly defined data flow at the outset of the project, or the eventual realization that the originally promised principle of “no data leaves the node” cannot be fully upheld during later stages of the project.

In the study, the interviewees often mentioned difficulties in assigning the GDPR roles of controller, joint controller or a processor and mapping related obligations to the various stakeholders involved in the federated network. While recent years have brought new guidelines from the European Data Protection Board (EDPB) (28) and judgments from the Court of Justice of the European Union (CJEU) (29–31), participants in the study noted that practical application of GDPR definitions remains unclear. One reason for these difficulties in the federated data networks is the involvement of multiple parties (data holders, users and the central operator), which undertake various data processing operations to ensure that federated networks function as a reliable source of research data. Data holders first establish a node, convert their datasets into the required data model, and make the transformed data available for analysis. Data users then query the data and submit requests to use it for their research purposes, with the actual research computations performed within the nodes. The central operator (also referred to as an aggregator or intermediary) (19) does not provide data nor use it for scientific purposes, however it plays a pivotal role in enabling federated data use by onboarding data holders and data users, facilitating interactions between them, participating in decisions concerning data harmonization and the establishment of common security requirements.

Earlier literature has already highlighted the difficulties of assessing GDPR roles in multi-partner consortia conducting biomedical research (8, 32). Previous papers examined, among other challenges, the definition of the aggregator's role and the distinction between joint controllers and other partners (12, 33). Against this background, our study provides empirical confirmation that these issues remain complex and contentious in practice, while also offering insights into potential solutions. Yet important gaps remain, as case-specific guidance (17) is scarce and federated repositories open to third-party researchers, which could provide benchmarks or best practices, are still being developed.

### 4.2 Data anonymization in federated networks: the impossible quest

Several interviewees raised the challenges of anonymizing personal data for scientific purposes—especially regarding unstructured or non-tabular health data, such as medical images, and genetic information—and academic literature also documents these difficulties extensively (34, 35). A recurring theme across these publications is the absence of concrete, standardized guidelines and benchmarks for effective data anonymization, both at the individual and aggregate levels (36). Moreover, the data protection community, regulators (37) as well as EU courts (38), continue to grapple with the fundamental question of interpreting the GDPR notion of personal data, especially in relation to the re-identifiability of pseudonymized information.

Our study contributes to this ongoing debate by highlighting the practical challenges of reaching a consensus on the status of shared data, particularly when the data can be assessed from different perspectives: that of the data holder, the intermediary, or the data user. Each of these stakeholders has different means to potentially re-identify the data subjects. Until greater legal clarity is achieved, the ambiguity surrounding whether data within a federated network qualifies as *personal data* under the GDPR significantly complicates the development of a robust contractual framework.

Participants also revisited the ongoing debate (39), about whether the current definition of personal data under the GDPR remains fit for purpose, or whether efforts to operationalize it into concrete, actionable guidance have either reached an impasse (40, 41). In response to these ambiguities, some participants have suggested the concepts such as *well-anonymized data* or *functionally anonymous data*. Those concepts that resonate with earlier academic literature (42) and the recent CJEU judgment in the case EDPS v SRB (38) and emphasize the contextual nature of identifiability, focusing on the actual ability of the data recipient to re-identify individuals, rather than on a universal assumption of identifiability.

Another important point raised by participants concerns the trade-off between data utility and the privacy of data subjects. As reported in earlier academic work, overzealous anonymization (43) can significantly diminish the accuracy of developed models and the validity of research outcomes (5), particularly when access to data is already indirect and constrained, as is often the case in federated approaches (15). This issue also feeds into a broader policy debate: an excessive focus on anonymization may divert resources and attention away from meaningful scientific research. Furthermore, there remains uncertainty as to whether policymakers fully grasp the inherent tension between enabling access to rich, high-quality data for general research purposes and ensuring the protection of individuals' privacy (40).

### 4.3 Data governance framework: “paradox at the heart of federated networks”

Our study demonstrates that concerns about insufficient harmonization of data access processes, which have been reported in the past (44), remain relevant in federated repositories, where authority over access is naturally spread across multiple actors. Our study highlights an intriguing paradox: data holders sign up to participate in the federated networks to retain control over the use of their data, yet for the federated networks to function efficiently for data users, the access process must be streamlined (45), necessitating that data holders relinquish a degree of that control. The challenges of agreeing on how to exercise data access control will remain relevant under the upcoming EHDS regulation. While the EHDS will establish a legal framework for secondary use of data and introduce standardized procedures for submitting access requests through health data access bodies, it will not override existing frameworks established by contractual or administrative arrangements between public and private entities [Article 1 (8) EHDS]. As a result, federated networks may still retain the ability to define their own governance rules applicable among participants.

Academic research has highlighted the evolving role of Data Access Committees (DACs) as an important topic of discussion (46). Our study shows that, beyond verifying compliance with dataset access conditions, DACs are increasingly expected to take on new responsibilities. One of them is assessing AI models to be trained on the data. This shift expands their mandate, requires additional expertise, and highlights the need for new tools to support their decision-making, for example by classifying data access requests according to privacy risk.

The study also reaffirmed earlier findings (47) that federated networks consist not only of data users and data holders, but also depend significantly on intermediaries (also known as aggregators or orchestrators). Participants emphasized the critical importance of the services provided by these intermediaries, which include maintaining a central data catalogue, facilitating user–holder interactions, onboarding and advising data nodes, enforcing security requirements, harmonizing access processes, optimizing repository data, and promoting research outcomes. While the scope of these services is broad, many projects lack a clearly designated party to assume this role after the end of the project term, and there is often reluctance to take on the associated responsibilities. Looking ahead, the legal role of orchestrators in federated networks warrants further exploration, particularly in light of emerging EU data governance rules, in particular on the data intermediation service providers (48) under the Data Governance Act (49) as well as trusted health data holders (Article 72 EHDS) or coordinators of databases from a various Member States organized into a single network of databases (Article 76.2 EHDS). These developments highlight a promising area for future research at the intersection of federated data governance, legal frameworks, and cross-border health data collaboration.

### 4.4 Limitations

This study is not without limitations. First, it relied on a qualitative design based on semi-structured interviews with a relatively small number of experts. Although interviews were conducted until data saturation was reached, the findings cannot be considered representative of all approaches to federated data sharing. The use of purposive and snowball sampling, while appropriate given the specificity of the target population, may have led to the inclusion of participants who are more visible or active in EU-funded projects, potentially limiting the diversity of perspectives.

Second, all interviews were conducted in English and online, which may have influenced participants' ability to fully express complex views and may have excluded experts less comfortable working in English. In addition, most interviews were conducted one-to-one, but one interview involved two participants from the same institution, which may have affected the dynamics of that discussion.

Third, data collection relied entirely on self-reported experiences and reflections of experts. No independent validation of statements was performed, and no observational or documentary data were collected. The study also did not aim to assess outcomes, effectiveness, or costs of federated data sharing solutions, but focused on experiences and perspectives related to their design and implementation.

Fourth, the research team was relatively small, and one researcher conducted all interviews. While transcripts were coded by both researchers to support analytical rigor, researcher interpretation may still have influenced data collection and analysis.

Fifth, the study was conducted within the context of EU regulatory frameworks, and the findings are therefore inherently EU focused. As a result, the outcomes cannot be directly transferred to other jurisdictions that operate under different legal and regulatory regimes.

Finally, although participants represented diverse professional backgrounds, the study does not capture variation within institutions, projects, or national contexts. As a result, the findings should be interpreted as providing in-depth insights rather than a comprehensive or generalizable account of federated data sharing practices. The conclusions reflect the views of a specific group of experts and are not intended to be statistically extrapolated to broader populations.

## 5 Conclusions

Our study does not discard the potential advantages of a federated approach, such as data minimization, however it also highlights the significant challenges of aligning federated networks architectures with data protection requirements. Notably, while federated networks may help initiate discussions about data sharing with new data holders, they do not offer a straightforward solution to legal and technical challenges of data sharing. The insights gained from this study can contribute to broader initiatives, such as the Data Spaces Support Centre (18) by informing the design of data governance models not only within the EHDS but also across other European data spaces that incorporate federated architectures (50).

## Data Availability

The datasets presented in this article are not readily available because the data of the participants cannot be disclosed. Requests to access the datasets should be directed to magdalena.kogutczarkowska@ugent.be.
