# Supplementary material for: Anonymization, accountability, and access: legal dimensions of health data sharing in federated networks. Perspectives from empirical study
Source: Front Digit Health. 2026 Mar 19;8:1719728. doi: 10.3389/fdgth.2026.1719728 (PMC13044123; doi:10.3389/fdgth.2026.1719728)
Supplement: Supplementary file 1 [file Datasheet1.pdf]

Supplementary Materials Manuscript 1719728

1) Overview of participant's expertise

Number	Area of expertise	Seniority
0.	Technical/ Infrastructure development	Midlevel
1.	Ethics expert	Senior
2.	Medical doctor	Senior
3.	Technical/Infrastructure development	Midlevel
4.	Technical/Infrastructure development	Midlevel
5.	Technical/AI developer	Midlevel
6.	Technical/Data scientist	Midlevel
7.	Project manager	Senior
8.	Technical/Data scientist	Senior
9.	Legal expert	Senior
10.	Project management	Senior
11.	Ethics expert	Senior
12.	Legal expert	Midlevel
13.	Technical/Data scientist/ Project management	Senior
14.	Legal expert	Midlevel
15.	Legal expert	Senior
16.	Legal expert	Senior
17.	Technical/Data scientist/ Project management	Senior
18.	Technical/Data scientist/ Project management	Senior

The experts were based in the following countries (alphabetically): Belgium, Canada, Finland, France, Greece, Norway, Spain, Spain, The Netherlands, UK, USA

2) <u>Inverview guide (Annex 1 to Form 'Request advice from FEC by researchers')</u>

<u>Before recording</u>

Good morning/afternoon. My name is [removed]. Like I mentioned in my email, I am a PhD candidate at Ghent University.

First, and before we get started, how are you doing today?

Great, another thing before we get started, I just want to explain what it is we are doing and the purpose of this interview. So, this interview is part of my PhD research in which I am looking into the use of federated repositories for sharing and re-using health data for scientific purposes. Within this project, we are closely looking at the strengths and weaknesses of the federated approach, privacy and data protection challenges and data governance practices and views on the upcoming EHDS regulation, considering the implications that it will have for the existing or planned federated repositories. For this reason, we would like to interview experts involved in development and deployment of these repositories to learn more about their views and experiences with federated repositories, their views on ensuring compliance with legal requirements and their attitudes towards the future EHDS.

As explained in the information letter I sent you, we will not use your name or any other identifying information and everything that you say will only be used for research purposes. We will be recording the interview, but the recording will be deleted after transcription. If you do not agree to recording, I will take notes from the interview. Please feel free to answer the questions openly and freely. There are no right or wrong answers.

Do you have any questions for me before we start?

Then if you agree, I will start the recording.

<u>Start recording</u>

<u>Interview guide</u>

<u>Introductory questions:</u>

Institutional and Professional Information

- Can you please introduce yourself and tell me how you would define your current role within your organization?

- How long have you been in this role?

Experiences and Perception of Using Federated Repositories

- What experience with participating in federated repositories for sharing health data with other researchers have you had?
- What were your expectations regarding sharing of health data in a federated repository?
- What are the strengths and successes you've observed when using federated data sharing approach when compared with the central one?
- What challenges and difficulties do you see when using federated repositories for health data sharing?

** "Federated repository": a network of multiple repositories (nodes) where the health data is stored by the data holders, which allows data users, mostly being researchers in the health field (such as developers of AI tools for healthcare), to conduct research using data from multiple repositories. Federated repository model is often contrasted with a centralized model, where the data is copied from the original location and stored in a central storage.*

<u>**Specific issues**</u> (please respond based on approach taken in the federated repository which you are familiar with):

Data protection and Privacy

- What are the privacy risks associated with using the federated repositories for sharing health data? In your view, what measures can be taken to mitigate those risks?
- What is your experience with addressing data protection requirements when sharing health data in federated repositories? Which GDPR requirements can be difficult for federated repositories to comply with?
- In your view, what elements of the federated repositories can safeguard the rights and freedoms of data subjects**? Which elements may impact rights and freedoms negatively?

***Rights and freedoms of data subjects can be understood both narrowly as referring to the right of protection of personal data, as well as more broadly, as stemming directly from the human rights law.*

Control over Data and Data Governance

- What is your experience with data governance (exercising control over access to data) in federated repositories? What works well? What is challenging?

- What in your view are the conditions for a compliant and successful use case of federated health data sharing and re-use?

- What is your experience with aligning the repository with the requirements of the Data Governance Act?

Quality of Data, Usefulness for AI Developers and Sustainability

- What are your views on whether federated repositories can assure accuracy and quality of research data?

- What are your views on whether federated repositories can be useful for AI developers?

- What you your views on whether federated repositories can be sustainable?

EHDS and Federated Repositories

- The EHDS proposal provides for a legal framework for sharing health data for secondary use. In your opinion, what will be the impact of EHDS on federated health data repositories? What will be challenging for federated repositories?

- Do you foresee that federated repositories will be needed when EHDS will be in force?

Concluding questions:

- Is there anything else you would like to add that we have not covered in this interview?
- Finally, do you know of any other expert that would be interested in discussing the topic during an interview?

Thank you for your time!

I will stop the recording now.

End recording

Interview Guide Checklist

1. Institutional and Professional Information

- Introduction of the interviewee and definition of their current role.
- Duration of the interviewee's current role.

2. Experiences and Perception of Using Federated Repositories

- Experience with federated repositories for sharing health data for research.
- Expectations regarding federated repositories
- Strengths and successes observed when using federated data sharing approach
- Challenges and difficulties when using federated data sharing approach

3. Data Protection and Privacy

- Privacy risks associated with using the federated repositories for sharing health data
    - Measures to mitigate those risks
- Experience with addressing data protection requirements in federated repositories
    - Examples of difficulties in ensuring GDPR compliance (if any)
- Opinion on safeguards to rights and freedoms of data subjects in federated repositories
    - Examples of negative impact on data subjects rights (if any)

4. Control over Data and Data Governance

- Experience with data governance in federated repositories
    - Examples that work well
    - Examples of challenges
- Opinion on conditions for a compliant and successful use case of federated health data sharing and re-use
- Experience with aligning the repository with the requirements of DGA

5. Quality of Data, Usefulness for AI Developers and Sustainability

- Evaluation of whether federated repositories can assure accuracy and quality of research data

- Opinion on federated repositories being useful for AI developers
- Opinion on federated approach being sustainable

6. EHDS and Federated Repositories

- Perspective on impact of EHDS on federated health data repositories
- Expectations regarding federated health data repositories being needed under EHDS

7. Concluding Questions

- Checking on additional comments which were not covered during the interview
- Other potential interviewees

3) <u>Overview of Identified Themes</u>

**Themes/subthemes**
**Legal aspects of setting up FN**
• „Data does not move" does not resolve all legal issues
○ Data is still being processed at FN
○ Need to work out who is processing data in FN
○ FN are complex/nuanced
• Difficulty in implementing GDPR/data protection laws
○ Applicability of GDPR to FN
▪ Placing the FN outside of GDPR
▪ FN does not change much under GDPR
▪ FN cannot help avoid GDPR
▪ Is access to data considered a transfer under GDPR?
○ Difficulty in assigning GDPR roles
▪ Joint controller agreement
▪ Sharing responsibility for data and breaches
▪ There exist different models in terms of controller/processor
▪ Operator of TRE is a controller
▪ Regulator guidance is needed
▪ Concerns about regulator guidance
○ Focus should be on the fundamental protection of the data subjects rights
▪ FN and impact on other fundamental rights
• Difficulty in understanding legal requirements
○ Fragmented implementation of GDPR in MS
○ Applying laws from many countries
○ Different legal requirements apply depending on the country
▪ MS Multiple regulatory frameworks
▪ Data comes from different countries
▪ Different laws apply to federated data
○ Need for legal experts locally to advise the nodes
○ Administrative and legal bottlenecks
• Preparing legal documentation
○ Need to ask for ethical and legal documents from the users
○ Partners need to demonstrate a legal basis
○ Documenting legitimate origin of data
○ Difficulties in obtaining ethical approvals
○ Ethics committees do not understand FN
○ Overpromising that data will not leave the node / that there will be no access to data
• Contractual set up
○ Trust and communication between the partners
▪ Risk aversiveness driving conversations about FN
▪ Conversations about comfort under GDPR

▪ Communication between legal and technical experts / Bridge between technical and legal aspects
▪ Challenges in communication between IT and legal
○ Challenges in formulating an agreement
▪ Long time to reach agreement/Time-consuming process of preparing legal documents
▪ Education and how we help researchers enter into these discussions
▪ Agreeing on the requirements and obligations of each partner
▪ Legal agreements on sharing data /<br>• Project agreement defines the permitted use of data<br>• Contracts defining the permitted use of data<br>• Defining the level of access through discussion between developers and data owners
▪ Preparing templates for data access
▪ Not many FN have already have a legal set up for a permanent setting
▪ Specific partners within projects with FL / Direct agreements between provider and user
▪ Divided responsibility
▪ Workspace provider for users /Similar solutions to secure processing environment
○ DPIA
• Other legal themes
○ Law not catching up to technical developments
○ Difficulty with complying with AI Act
○ Existing AI assessment frameworks are not aligned with FN
○ Can data be exchanged for free products?
○ Data Act
○ Data Governance Act
▪ Data Governance Act is creating problems
▪ Hype about data altruism
○ Ownership/IP rights to the outputs from the data<br>○ Less protectionism over data<br>○ Commercial companies access arrangements<br>○ Less protectionism around data
○ Requirements of pharmacovigilance
○ MDR requirements in FN
○ Similar local legislations to EHDS
**Data anonymization**
• Individual level data in the nodes needs to be de-identified
○ Nodes need to contain individual level data
○ Data is rarely fully anonymized
• Balancing usability and privacy of data
○ Cost/trade-off of privacy of the data
○ Perfectly anonymous data is useless

• When is data anonymous?
○ Unclear understanding of the anonymization/problems with anonymization terminology<br>○ Controllers have different views on data anonymization<br>○ Lack of common understanding at which point the data is anonymous/
○ Problems with anonymization of certain types of data, e.g. images
○ More flexibility in the Uk regarding status of data
○ Midway category of data
○ Data "functionally anonymous"
○ Concept of functionally anonymous data enables international collaboration
○ Data „anonymous in context" / Taking into account the environment when assessing if the data is anonymous
○ Concept of "well anonymized data"
○ Regulator should engage and understand FN to give detailed guidance that is practical
○ More risk if the data includes genetic data
• Data anonymization issues specific to FN
○ The results from the nodes should be anonymous
○ FN can decrease re-identification risk if the data is „well anonymized"
○ Risk of re-identification of individual level data v. Statistical data
○ Different scenarios of FN
▪ Best privacy scenario if the researchers do not see the anonymized data<br>▪ Best privacy scenario is often not practically feasible for the researchers
○ Local constraints
○ FN bring many experts together and have more expertise for data anonymization
○ FN provide anonymization tools / A need to optimise data security
○ Identifiability of data when linking data points
○ Anonymization of data in the nodes and anonymization of results returned to the central aggregator
○ FN help data anonymization?
**Data governance**
• Challenges in setting up governance model in FN
○ Decisions on governance are pushed till the end
○ Tension between nodes having control over data access and streamlining access process via DAC
○ Difficult communication between multiple partners
○ Data governance is more complex
○ Technical complexities are less than human complexities
○ Mixed models/approaches
○ Data governance when the data comes from several hospitals/providers

○ De-centralised data access
○ Different levels of federation
○ Different models of governance of access request exist / Staged approach
○ Role of an intermediary in the repository<br>○ Role of central aggregator
○ A centralised data governance support
• Governance and control over data stays with data providers
○ Decisions on data access stay will the controller (node)
○ Holders have control over who uses the data
○ FN expand the role of the data providers<br>○ Understanding of control over data
○ Different limits/ conditions on data use depending on the hospital/provider<br>○ Data access requirements should fit
○ Controllers/nodes decide which results they release to researcher
○ Obtaining feedback to the request from all the hospitals
○ Obtaining feedback to the request from all the hospitals/providers
○ Difficulties if the decision stays with the node
○ Obtaining multiple permissions from the controllers is unsustainable
○ Differences in approach from involved partners / Diversity of approaches
○ Streamlining the data governance when decided by data holders
○ FN members have to agree to change of the protocol of a study
○ Allowing data download requires trust between researcher and data provider
• Delegating decisions on data access to DAC
○ Lead controller sets up data access committee
○ Composition of data access committee<br>○ Representativeness from different data controllers, public and patient representatives
• Changing role of DACs
○ Data Access committee checking the AI models
○ DAC can react quickly to data access applications
• Tools/methods for streamlining the data governance in FN
○ Success of federated approach depends on streamlining governance, data protection ethics
○ Having a clear data protocol<br>○ Importance of data annotation/metadata
○ Different limits/ conditions of governance depending on the case<br>○ Different track of governance depending on the type of request<br>○ Risk stratification approach for data access requests
○ Data access/Granularity of data access/ A more nuanced process
○ Tools for selecting the permissions
○ Contracts defining the permitted use of data

○ Pre-set levels of granularity on the use of data which is permitted
• Other important governance observations
○ Lack of information about actual governance models
○ Promoting research results obtained thanks to FN
○ User should acknowledge the source of data
○ User should upfront understand restrictions before asking for access<br>○ Similarities between federated and central model
○ Setting up governance model for after the project ends
